# Trimethylamine N-Oxide Promotes Cell Proliferation and Angiogenesis in Colorectal Cancer

**DOI:** 10.1155/2022/7043856

**Published:** 2022-07-04

**Authors:** Shuyan Yang, Hui Dai, Yimei Lu, Rui Li, Chengjin Gao, Shuming Pan

**Affiliations:** ^1^Department of Radiology, Xinhua Hospital, Shanghai Jiao Tong University School of Medicine, Shanghai 200092, China; ^2^Department of Emergency, Xinhua Hospital, Shanghai Jiao Tong University School of Medicine, Shanghai 200092, China

## Abstract

*Background*. Of all intestinal microbiome-derived metabolites, trimethylamine N-oxide (TMAO) has received increasing attention because of its potent role in colorectal cancer development. Accumulating evidence suggests that TMAO generated by the gut microbiota is a new and important player in the etiological process of colorectal cancer. Nevertheless, the carcinogenic mechanism of TMAO in colorectal cancer remains unclear. In this study, TMAO induced colorectal cancer cell proliferation and produced higher vascular endothelial growth factor A (VEGFA) levels *in vitro*. *In vivo*, after long-term choline feeding in tumor-bearing mice, circulating TMAO levels, tumor volume, new blood vessel formation, and VEGFA and CD31 amounts were increased significantly. This study revealed that TMAO exerts oncogenic effects by promoting cell proliferation and angiogenesis in colorectal cancer.

## 1. Introduction

Colorectal cancer (CRC) ranks third among all malignancies worldwide and represents the second deadliest malignancy. In 2020, >1.9 million incident CRC cases and 935,000 deaths were expected, representing 10% of call cancer-related deaths [1]. This incidence rate is approximately 4 times higher in transition countries than in nontransition countries. It is widely believed that diet plays an important role in CRC development. Excessive red meat and high fat consumption affects gut microbiota composition, producing metabolites that cause intestinal inflammation, creating the initial carcinogenic environment for CRC development [2].

Dietary choline and L-carnitine, found in large amounts in red meat, are metabolized by the microbiota when they reach the gut, producing trimethylamine (TMA). After absorption, TMA enters the liver through the portal vein, where it undergoes oxidation into trimethylamine N-oxide (TMAO) by flavin-containing monooxygenases (FMOs), mainly FMO3. TMAO is next released into the circulatory system with eventual renal elimination [3–5]. TMAO is involved in multiple diseases, including atherosclerosis, diabetes, and malignant diseases. A genome-wide analysis demonstrated that TMAO is genetically related to CRC, with TMAO-associated and CRC-associated genes sharing gene pathways involved in immunity and the cell cycle and so on [6]. TMAO might represent an important intermediate marker associated with colorectal cancer carcinogenesis, dietary meat and fat, and intestinal microbiome. In addition, TMAO is involved in the progression and etiology of CRC and thus might be relevant to CRC diagnosis, prognosis, and therapy [7–10]. Although a possible association of TMAO with CRC has been proposed, there is no direct evidence for TMAO involvement in CRC.

We report the effects of high TMAO levels on the CRC cells *in vitro* and *in vivo*. CRC occurrence and development are closely related to tumor angiogenesis. We found that TMAO enhanced the secretion of vascular endothelial growth factor A (VEGFA) by CRC HCT-116 cells and promoted tumor proliferation. The present work was aimed at examining TMAO's effect in CRC development and at providing novel insights into TMAO's role in colorectal cancer.

## 2. Materials and Methods

### 2.1. Cells and Cell Treatment

Human CRC HCT-116 cells were provided by the Chinese Academy of Sciences Committee Type Culture Collection Cell Bank (China). The HCT-116 cell culture was routinely maintained in RPMI 1640 (Gibco, USA) containing 10% fetal bovine serum (Gibco, USA) and 1% penicillin/streptomycin (Gibco, USA). When the cells reached 80-90% confluence, cell passaging was carried out with 0.25% trypsin/EDTA (Gibco, USA), and the cells were seeded for experiments. Periodic testing ensured the absence of mycoplasma contamination. In the *in vitro* study, the HCT-116 cells were randomized into the control (Con, RPMI 1640) and TMAO (50, 250 and 500 *μ*M) groups. After treatment, the cells and culture supernatants were collected.

### 2.2. Cell Proliferation Assay

CCK-8 (Beyotime, China) was utilized for assessing cell proliferation. In brief, the cells underwent seeding in 96-well plates at 10^4^/well followed by a 24 h incubation. Different TMAO concentrations were administered to the HCT-116 cells for 24 h. Following treatment, 10 *μ*l/well of the CCK-8 solution was supplemented for 3 h at 37°C, and optical density was read at 450 nm with a microplate spectrophotometer. Every group was set for five duplicate wells.

### 2.3. Cell Apoptosis Assay

Apoptotic rates were determined flow cytometrically with the apoptosis detection kit (BD, USA), as directed by the manufacturer. The cells were plated in 6-well plates at 5 × 10^6^/ml. Upon treatment with TMAO, the cells underwent washing once with binding buffer. Then, FITC and PI stains were added to each well for 15 min at ambient, shielded from light. Data were assessed with Flow Jo 7.0.

### 2.4. Enzyme-Linked Immunosorbent Assay (ELISA)

VEGFA levels in the cells and culture supernatants were detected with the ELISA VEGFA kit (Ruifan, RF1092) as directed by the manufacturer.

### 2.5. Animals

This study was performed according to a protocol approved by the Ethics Committee of Xinhua Hospital Affiliated to Shanghai Jiao Tong University School of Medicine. Sixty 5-week-old male BALB/c nude mice were provided by Shanghai Slac Laboratory Animal Co., Ltd. The right hind flank of each mouse was injected subcutaneously with 0.2 ml PBS containing 10^7^ cells. Mice were provided acidified water with/without 1.3% choline (Sigma, USA) for 4 weeks from implantation [11]. After treatment, the animals underwent fasting for 4 h prior to blood and tumor tissue sample collection. Tumor volume was determined as *V* = 1/2 × length × width^2^ at 7-day intervals for 28 consecutive days, alongside body weight measurements. For terminal experiments, the animals underwent euthanasia by CO_2_ exposure.

### 2.6. Histology Examination

Tumor tissues in each group underwent formalin fixation, paraffin embedding, and 4 *μ*m sectioning. Then, hematoxylin and eosin (H&E), Ki67 antibody (Abcam, ab15580), VEGFA antibody (Proteintech, 19003-1-AP) and CD31 antibody (Abcam, ab182981) staining, and the terminal deoxynucleotidyl transferase dUTP nick end labeling (TUNEL) (Beyotime, C1091) staining were carried out, respectively. VEGFA and CD31 signals were assessed using a digital image analysis system (Moti Med 6.0), and the area densities of positive targets were determined.

### 2.7. Measurement of TMAO

BioNovoGene performed TMAO detection. Plasma TMAO concentrations were measured by high-performance liquid chromatography.

### 2.8. Data Analysis

SPSS 20.0 (SPSS Inc., USA) was used for data analysis. Quantitative data were compared by the *t*-test and one-way ANOVA for group pairs and multiple groups, respectively. In case of unequal variance, the rank-sum test was performed. Categorical data were assessed by the chi-square test. *P* < 0.05 indicated statistical significance.

## 3. Results

### 3.1. TMAO Induces Cell Proliferation but Has No Effect on Apoptosis

**Figure 1:**
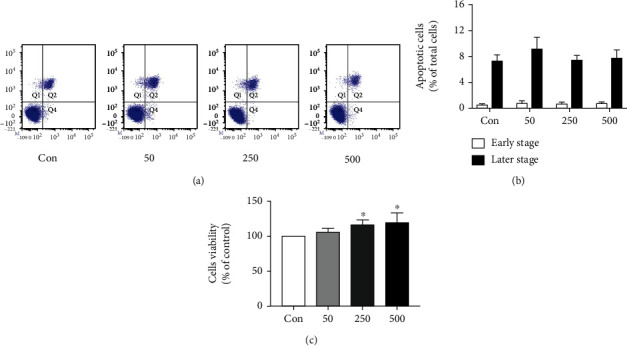
Effects of TMAO on proliferation and apoptosis in the HCT-116 cells. (a) Cell apoptosis assessed by flow cytometry. (b) Apoptotic rates in various groups. (c) Cell proliferation assessed with CCK-8. ^∗^*P* < 0.05 versus Con group (*n* = 5).

Considering that the excessive proliferation of tumor cells is one of the pathways of tumorigenesis, we preliminarily investigated the effect of different concentrations of TMAO on the proliferation of HCT-116 cells. As shown in Figure 1(c), the CCK-8 assay demonstrated elevated cell viability in the TMAO group compared with control cells, in a dose-dependent fashion. We next assessed whether TMAO affects apoptosis. Compared with the control group, there were no significant differences in the TMAO groups (Figures 1(a) and 1(b)). These results preliminarily indicated that TMAO treatment significantly enhanced cell proliferation without affecting apoptosis.

### 3.2. TMAO Enhances VEGFA Secretion in the HCT-116 Cells

**Figure 2:**
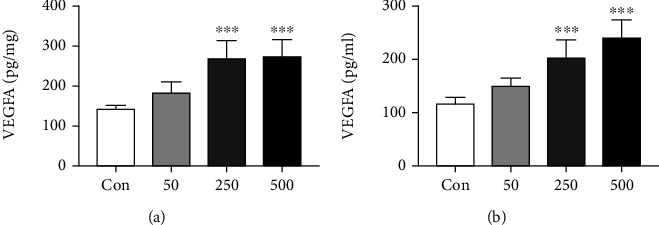
VEGFA expression in the HCT-116 (a) cells and (b) cell supernatants after incubation with different concentrations of TMAO. ^∗∗∗^*P* < 0.001 versus Con group (*n* = 5).

Angiogenesis is an important prerequisite for tumor proliferation, migration, and invasion. VEGFA plays a key role in angiogenesis in CRC and has become a major target of antiangiogenic drugs. Therefore, VEGFA levels in the cells and cell supernatants were assessed. The results showed increased VEGFA secretion after TMAO treatment, in a concentration-dependent fashion (Figures 2(a) and 2(b)).

### 3.3. Chronic Choline Feeding Increases the Tumor Volume in Tumor-Bearing Mice

**Figure 3:**
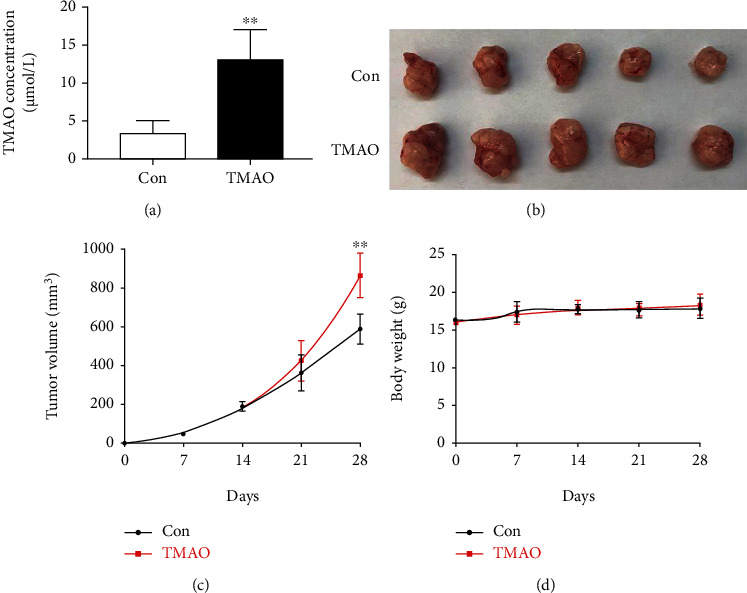
Tumor volumes and body weights in tumor-bearing mice after choline treatment. (a) Plasma levels of TMAO. (b) Representative photographs of exfoliated tumors from tumor-bearing animals. (c) Tumor volume curves and (d) body weight growth curves of mice over time in various groups. Con: control group; TMAO: 1.3% choline feeding. ^∗∗^*P* < 0.01 versus Con group (*n* = 5).

Given the role of TMAO in the HCT-116 cells, we examined whether the effects observed *in vitro* could be recapitulated by long-term choline feeding. Tumor-bearing mice were provided chemically with defined water containing 1.3% choline or not. After 4 weeks of drinking, blood TMAO levels were markedly increased (Figure 3(a)). In comparison with control animals, tumor volumes in choline-fed mice were markedly increased (866.01 ± 115.99 mm^3^ vs. 590.11 ± 76.52 mm^3^), while body weights were comparable in both groups (Figures 3(b)–3(d)).

### 3.4. Chronic Choline Feeding Promotes Tumor Proliferation in Tumor-Bearing Mice

**Figure 4:**
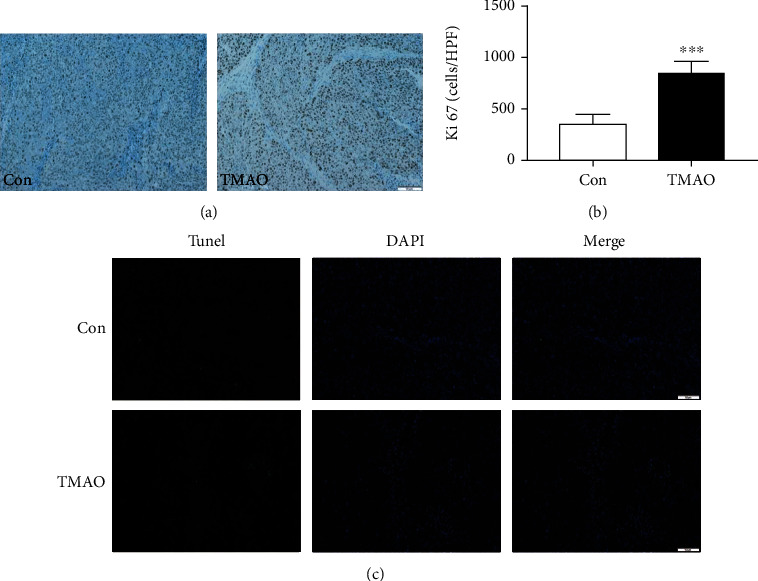
Choline treatment alters proliferation and apoptosis in tumor-bearing mice. (a) Immunohistochemical staining for Ki67 in tumors from implanted mice in both groups and (b) quantitative analysis. (c) TUNEL staining of tumor tissues. Con: control group; TMAO: 1.3% choline feeding. ^∗∗∗^*P* < 0.001 versus Con group (*n* = 5).

Ki67 expression is broadly utilized in clinic to evaluate the proliferative activity of tumor cells. The proportion of Ki67-positive tumor cells often correlates with the clinical course of colorectal cancer. The results demonstrated that Ki67 expression was markedly higher in tumor tissue specimens from mice treated with choline compared with the control group (Figures 4(a) and 4(b)), while apoptosis in the tumor tissue was unaltered by choline (Figure 4(c)).

### 3.5. Chronic Choline Feeding Promotes Tumor Angiogenesis

**Figure 5:**
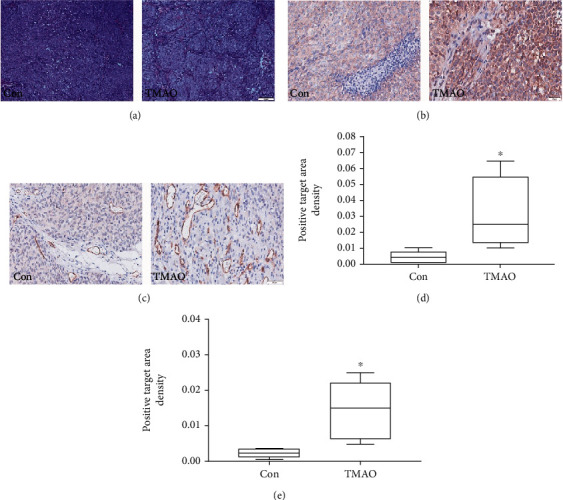
H&E-stained sections and immunohistochemical staining for VEGFA and CD31 in tumor specimens from tumor-bearing animals. (a–c) Representative micrographs of H&E, VEGFA, and CD31 staining. (d–e) Quantitative analysis of VEGFA and CD31 levels. Con: control group; TMAO: 1.3% choline feeding. ^∗^*P* < 0.05 versus Con group (*n* = 5).

To further assess TMAO's effect on tumor angiogenesis *in vivo*, we first performed H&E staining of tumor tissue specimens from the two groups. We found that tumor-bearing mice in the TMAO group had increased tumor angiogenesis (Figure 5(a)). Next, immunohistochemical staining revealed that VEGFA and CD31 amounts were significantly elevated in the TMAO group compared with control animals (Figures 5(b)–5(e)).

## 4. Discussion

TMAO, an oxidative derivative of dietary choline, can be catabolized by the gut microbiota. TMAO might constitute a critical factor linking diet, gut microbiota, and diseases. Current advances in research have supported the notion that elevated TMAO content is an important biomarker of or an independent risk factor for multiple adverse disorders [12–18].

Numerous reports have focused on the associations of elevated blood TMAO with cancers, including prostate [19], oral [20], breast [21], and primary liver [22] cancers. Interestingly, reports indicated that TMAO is positively correlated with CRC. For example, Bae et al. [23] demonstrated a positive correlation between plasma TMAO and colorectal cancer risk in postmenopausal women in the United States. In addition, X. Liu et al. reported high serum TMAO levels as a good indicator of poor prognosis and low disease-free survival in CRC patients [24]. However, the published data were based on blood samples from CRC patients, and how TMAO affects CRC remains unclear.

In this study, we initially treated the HCT-116 cells with various concentrations of TMAO, which enhanced the proliferative ability of these cells as well as VEGFA production. In addition, CRC tumor-bearing mice were provided drinking water containing 1.3% choline for 4 weeks. The results revealed that circulating TMAO content in the choline group was significantly increased compared with control values, which also verified that a choline-rich diet increases TMAO levels. Meanwhile, tumor volumes in mice increased as well as tumor neovascularization and VEGFA and CD31 amounts.

Angiogenesis is critical for the growth and progression of solid tumors. Previous findings have shown microvessel density (MVD) is positively correlated with invasion depth, lymph node and distant metastases, and TNM stage in CRC patients [25]. CD31, a sensitive and specific endothelial marker of MVD, has positive correlations with TNM stage and lymph node metastasis [26]. Meanwhile, VEGFA, a major proangiogenic factor, induces mitosis and regulates endothelial cell permeability. VEGFA secreted by tumor cells and the surrounding stroma induces endothelial cell proliferation, enhancing angiogenesis [27]. Furthermore, increased VEGFA amounts were shown to be significantly correlated with advanced lymph node and distant metastases [28]. As demonstrated above, TMAO upregulated VEGFA and CD31. We speculate that TMAO may exert a tumor-promoting effect by promoting tumor angiogenesis.

Previous studies have shown that the aorta of LDLR^−^/^−^ mice has increased expression of inflammatory genes after receiving choline in the diet. Meanwhile, injection of physiological levels of TMAO induced similar inflammatory changes and activated the mitogen-activated protein kinase (MAPK), extracellular signal-related kinase, and nuclear factor-*κ*B (NF-*κ*B) pathways [11]. The above findings have not been confirmed in CRC. NF-*κ*B signaling regulates tumor angiogenesis and invasiveness, and we hypothesized that TMAO may induce angiogenesis through NF-*κ*B signaling.

Here, we only preliminarily studied TMAO's role in colorectal cancer cells, and the specific mechanism was not elucidated. Inflammation, oxidative stress, DNA damage, and protein misfolding may represent potential events linking TMAO to carcinogenesis [29–31]. These findings may provide a theoretical basis for further studying the specific mechanism by which TMAO affects colorectal cancer. At the same time, it may be interesting to develop diagnostic methods detecting TMAO in human fluids, including urine [32]. In a recent study, Lakshmi et al. designed a sensor to measure TMAO levels in real urine specimens [33]. The mechanism of TMAO's effects in colorectal cancer is being further explored by our team.

## Data Availability

The data used to support the findings of this study are available from the corresponding author on reasonable request.
